# Arterial spin labeling characterization of cerebral perfusion during normal maturation from late childhood into adulthood: normal ‘reference range' values and their use in clinical studies

**DOI:** 10.1038/jcbfm.2014.17

**Published:** 2014-02-05

**Authors:** Patrick W Hales, Jamie M Kawadler, Sarah E Aylett, Fenella J Kirkham, Christopher A Clark

**Affiliations:** 1 Imaging and Biophysics Unit, Institute of Child Health, University College London, London, UK; 2 Neurosciences Department, Great Ormond Street Hospital, London, UK; 3 Neurosciences Unit, Institute of Child Health, University College London, London, UK

**Keywords:** ASL, arterial spin labeling, brain imaging, cerebral hemodynamics, cerebral blood flow measurement, magnetic resonance, MRI, perfusion weighted MRI

## Abstract

The human brain changes structurally and functionally during adolescence, with associated alterations in cerebral perfusion. We performed dynamic arterial spin labeling (ASL) magnetic resonance imaging in healthy subjects between 8 and 32 years of age, to investigate changes in cerebral hemodynamics during normal development. In addition, an inversion recovery sequence allowed quantification of changes in longitudinal relaxation time (*T*_1_) and equilibrium longitudinal magnetization (*M*_0_). We present mean and reference ranges for normal values of *T*_1_, *M*_0_, cerebral blood flow (CBF), bolus arrival time, and bolus duration in cortical gray matter, to provide a tool for identifying age-matched perfusion abnormalities in this age range in clinical studies. Cerebral blood flow and *T*_1_ relaxation times were negatively correlated with age, without gender or hemisphere differences. The same was true for *M*_0_ anteriorly, but posteriorly, males but not females showed a significant decline in *M*_0_ with increasing age. Two examples of the clinical utility of these data in identifying age-matched perfusion abnormalities, in Sturge–Weber syndrome and sickle cell anemia, are illustrated.

## Introduction

The transition from childhood, through adolescence and into adulthood, marks a period of structural and functional change in the normal development of the human brain. The cerebral cortex undergoes its most dynamic structural change in adolescence,^1^ and previous studies have indicated that this is accompanied by a further rapid change in cerebral perfusion (subsequent to the dynamic changes that occur in the first year of life), which occurs between the ages of 10 to 20 years.^2^

The nonionizing nature of magnetic resonance imaging (MRI), coupled with its superior spatial resolution, makes it an attractive technique for studying the developing physiology of the brain. Although metabolism is not measured directly with MRI, cerebral perfusion can be quantified, which under physiologic steady-state conditions is coupled to the level of cerebral oxygen and glucose consumption,^3^ and as such mirrors the brain's metabolic demand and neuronal function.

Traditionally, MRI-based perfusion techniques have involved serial imaging after the injection of a paramagnetic contrast agent, to dynamically track the passage of a labeled bolus through the vasculature. However, the injection of an exogenous contrast agent limits the applicability of these techniques in longitudinal studies, and precludes their use in patients with renal failure, due to the associated risk of nephrogenic systemic fibrosis.^4^ Its application in children has also been limited due to reduced patient comfort, and technical difficulties in administering the intravenous contrast agent.

Arterial spin labeling (ASL) is an emerging alternative MRI technique for fully noninvasive quantification of cerebral blood flow (CBF).^5, 6^ Although the signal-to-noise ratio (SNR) is inherently lower than measurements acquired using injected paramagnetic contrast agents, ASL has the advantage that it uses an endogenous tracer, by magnetically labeling water in the arterial blood supply. As such, it is a completely noninvasive and nonionizing technique, allowing for safe repeated measurements and increased patient comfort. Furthermore, ASL provides measurements of CBF in fully quantitative physiological units (such as mL blood/100 g tissue per minute), and as such is well suited for use in longitudinal or multicenter studies. These combined benefits have seen ASL move from the field of research into routine clinical practice in recent years.^7, 8^

### Arterial Spin Labeling Perfusion Quantification

Perfusion quantification using ASL is performed by inverting the longitudinal magnetization of the arterial blood flowing into the tissue, waiting for a given ‘inflow time' (TI), then acquiring an image (known as the ‘*label*' acquisition). The same process is repeated without labeling the inflowing arterial blood (the ‘*control*' acquisition), after which the perfusion information is contained in the subtraction between the two acquisitions (d*M*, in which d*M*=*control−label* signal intensity).

A source of potential systematic error when estimating CBF using ASL is the delay between the application of the label and the arrival of the labeled bolus of blood at the imaging voxel (known as the bolus arrival time, BAT). Spatial variations in BAT will result in a variable amount of tagged blood having been delivered at the time of the acquisition (TI), and failure to account for this can lead to significant errors in CBF quantification.

A second source of error is the ‘through-flow' effect, which occurs when the acquisition is made at an early TI, leading to an overestimation in CBF, as the d*M* signal includes a significant contribution from intravascular tagged blood which is destined to perfuse more distal tissue. Both the above problems are linked to the fundamental issue that the time required for labeled blood to travel from the tagging region to the capillary bed is both spatially variable and generally comparable to the *T*_1_ relaxation time of blood, ensuring that multiple dynamic processes are occurring at the time of acquisition (delivery, exchange, clearance by *T*_1_ relaxation, and flow).^9^

A simplified kinetic model was introduced by Buxton *et al*^10^ to account for some of these processes, in which the time-dependent difference signal (for pulsed-ASL) is given by:


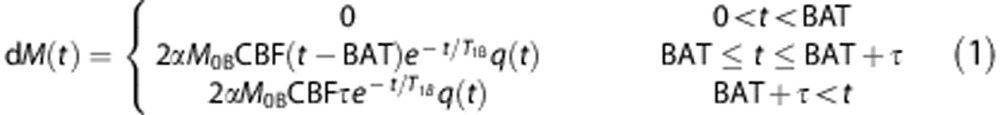


where


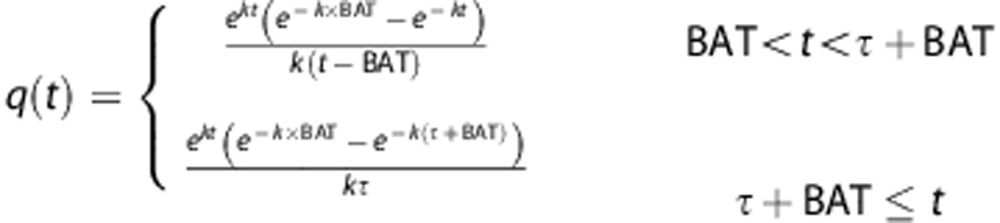


and


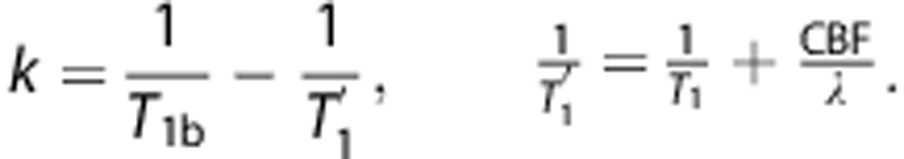


Here, *α*=inversion efficiency, *M*_0B_=equilibrium longitudinal magnetization of blood, *T*_1B_=longitudinal relaxation time of blood, *T*_1_=longitudinal relaxation time of tissue, *λ*=equilibrium tissue/blood partition coefficient of water, and *T*=bolus duration.

If measurements are made at multiple TIs, then the d*M* time series can be fit to equation (1), with BAT, CBF, and *T* as fitted parameters. However, due to SNR and scan time constraints, the majority of clinical studies currently acquire ASL data at a single TI. As shown in equation (1), the amount of labeled blood entering a voxel will increase linearly between BAT≤*t*≤BAT+*T*, and as such, the optimal TI should be ≥BAT+*T*, to eliminate the effect of transit time on CBF quantification. A challenge when designing an ASL protocol is that the delay required for this is not generally known *a priori*. To help counteract this, it has been shown that ‘Q2TIPS'^11^ saturation pulses can be applied after a given delay (TI_1_) after the labeling, to ‘cut-off' the trailing end of bolus, ensuring a well-defined bolus width leaves the labeling region. However, the value of *T* will also be influenced by bolus dispersion between the labeling region and the imaging voxel, which varies as a function of vessel size, length, and tortuosity. As such, it would be advantageous to know what starting values and local variations in BAT and T to expect when designing a single-TI ASL protocol.

A further point to note is that, for accurate quantification of CBF, *M*_0B_, *T*_1B_, and *T*_1_ must be known. A series of inversion recovery acquisitions (with varying TIs) can be performed to measure *T*_1_ and *M*_0_ on a voxelwise basis. From this, *M*_0B_ can be determined, using *M*_0B_=*M*_0_/*λ*^12^ (note calculating this on a voxelwise basis accounts for radio frequency coil B_1_ inhomogeneities). Due to difficulties in locating a voxel filled purely with blood, literature values of T_1B_ are generally used, which in studies involving children and young adults, should be adjusted for age and sex.^13, 14^ However, in many previous studies, the ‘*q*(*t*)' term in equation (1) has been assumed to be unity, and the effects of both the venous clearance and the exchange of labeled blood water into the surrounding tissue have been ignored. As the clearance of tagged water by flow is much slower than the rate of delivery, the former assumption is generally valid in the healthy human brain. However, it has been shown that exchange between capillaries and tissue appears to occur at TIs >1,000 ms after the application of the tag.^15^ As single-TI measurements are generally made at TI>1,000 ms, the magnitude of the error introduced by the latter assumption will depend on the difference between *T*_1B_ and the local *T*_1_ value. In normal gray matter (GM), this is generally small; however, in white matter and under certain pathologies, this error can be significant. Also, *T*_1_ in cortical GM is known to vary with age,^16^ and as such failure to account for this will lead to a variable, age-dependent error in CBF quantification.

Due to the rapid alterations in CBF that occur during the transition from late childhood into adulthood,^2^ it is important that age-related changes in both the dynamic ASL signal and the *T*_1_ and *M*_0_ values used to calculate CBF are fully understood in this age group. Although a number of studies have been published in which ASL has been used to measure cerebral perfusion as a function of age, the majority of these only report CBF values, generally because the data were acquired with either a single or a few TIs,^2, 17, 18, 19^ and as such the changes in the dynamic ASL signal (and associated changes in BAT and *T*) have not been extensively studied, particularly in children and young adults. In this study, we investigated how *T*_1_, *M*_0_, and hemodynamic properties of the dynamic ASL signal vary as a function of age, in healthy subjects between 8 and 32 years of age. The lower limit of this age group was chosen to ensure all subjects could be scanned without the use of sedation, as different kinds of sedation may modify CBF in different ways,^20^ and previous studies have shown significant increases in CBF in halogen-sedated compared with awake children.^2^ We provide ‘reference range' limits (see Materials and Methods) for normal values in both raw ASL data and fitted parameters for nonsedated subjects, to be used both as a tool in future clinical studies and when designing an optimized single-TI ASL protocol. We also show two clinical examples of how these reference range values can be used to identify age-matched perfusion abnormalities: in one patient with Sturge–Weber syndrome and one patient with sickle cell anemia (SCA).

## Materials and methods

### Subjects

Approval to conduct this study was obtained from the Research Ethics Committee at Great Ormond Street Hospital. All healthy subjects provided written informed consent, in accordance with our institutional ethical review board. A total of 30 healthy subjects (7 males and 9 females below 18 years, 7 males and 7 females above 18 years) were studied, aged between 8 and 32 years (mean 18.5 years). In addition, ASL was performed in conjunction with the standard clinical imaging protocols in use in our institution in two patients. The first was a 17-year-old Sturge–Weber patient with a right pial angioma, who was experiencing symptomatic focal epilepsy, migraine headache, learning difficulties, and intracranial hypertension. The second was a 12-year-old patient with homozygous SCA with no evidence of neurologic abnormality seen on T2-weighted MRI. Blood taken in this patient 6 weeks before MRI revealed a hemoglobin level of 8.6 g/dL (normal range: 11.5 to 15.5 g/dL) and hematocrit of 23% (normal range: 35% to 45%). Peripheral blood oxygen saturation was taken as 96% on the day of MRI.

### Magnetic Resonance Imaging

All experiments were performed on a 1.5-T Siemens Magnetom Avanto scanner (Siemens, Erlangen, Germany), equipped with 40 mT/m gradients and a 12 channel head receive coil. Arterial spin labeling data were acquired using a flow-sensitive alternating inversion recovery pulsed-ASL sequence, with 3D single shot GRASE data acquisition,^21^ with the following imaging parameters: repetition time=3.0 seconds, echo time=31.6 ms, 8 averages, field of view=230 mm, matrix size=64 × 64, 20 contiguous slices with 5 mm thickness. Measurements were made at six TIs, ranging from 0.2 to 2.2 seconds in 0.4-second intervals, with a total scan time of 4.8 minutes. Background suppression of static tissue was used, as described by Günther *et al*,^21^ with × 10 scanner gain, to maximize the signal from the inflowing blood. A T_1_-mapping sequence was then performed in each subject, comprising a series of inversion recovery acquisitions (TI=0.2, 0.6, 1.4, and 2.4 seconds) with identical readout, field of view and resolution as the ASL sequence, but without the use of background suppression.

### Postprocessing

All data analysis was performed using Matlab (MathWorks Inc., Natick, MA, USA), and all model fitting was performed using an iterative Nelder–Mead nonlinear least squares algorithm.

**Figure 1:**
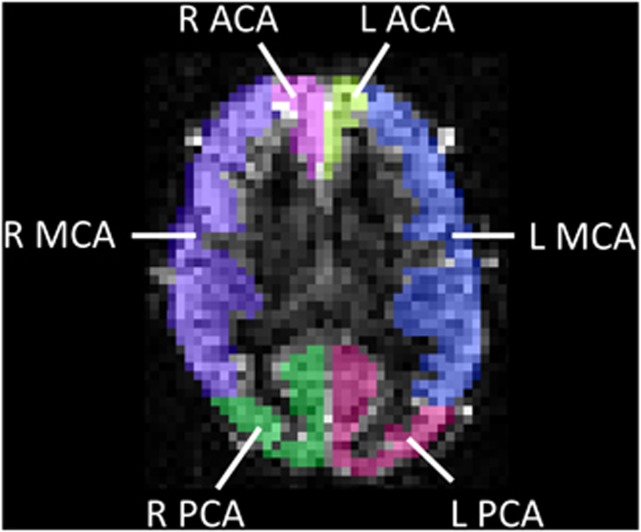
Example of arterial spin labeling (ASL) image segmentation, based on vascular flow territories. An axial ASL difference image (d*M*) is shown, at inflow time (TI)=1.8 seconds, with a semitransparent overlay illustrating six vascular territories. L, R=left, right; ACA, MCA, PCA=anterior-, middle-, and posterior-cerebral arterial flow territories, respectively.

First, the GM in the ASL and T_1_-mapping images was segmented into major vascular territories, using the flow territory templates established by Tatu *et al.*^22^ Following these templates, regions of interest (ROIs) defining the left and right cortical vascular territories of the anterior cerebral artery (ACA), middle cerebral artery (MCA), and posterior cerebral artery (PCA) were drawn on a standard MNI (Montreal Neurological Institute) template. These were then registered onto each subject's ASL/*T*_1_-mapping images, using an affine 12 parameter model provided by the FLIRT algorithm in FSL (FMRIB's Software Library, Oxford University, www.fmrib.ox.ac.uk/fsl). These ROIs were manually corrected in each subject, where necessary, to correct for registration errors caused by the point spread function inherent in the 3D GRASE acquisition. An example from a representative subject is shown in Figure 1.

After image segmentation, regional values of *T*_1_ and *M*_0_ were calculated by fitting the mean signal intensity from the *T*_1_-mapping acquisition in each ROI to the following equation:


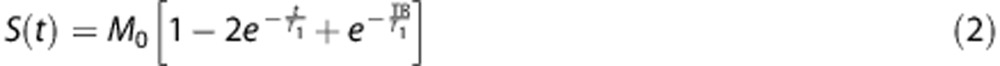


with *T*_1_ and *M*_0_ as fitted parameters. A mean value of *M*_0B_ was then calculated in each ROI, using *M*_0B_=*M*_0_/*λ*, with *λ*=0.9.^12^ After this, the mean ASL d*M*(*t*) signal from each ROI was fit to equation (1), using the calculated values of *T*_1_ and *M*_0B_, and assuming *α*=1. Age- and gender-corrected values of *T*_1B_ (at 1.5 T) were used for each subject, based on the data presented in Piechnik *et al.*^23^ This provided fitted values of BAT, CBF, and *τ* in each ROI. In addition, both the peak value of the fitted d*M* curve (d*M*_max_) and time at which the peak occurred (TI_max_) were recorded.

### Reference Range Regions

Using the method described by Royston,^24^ data from all healthy subjects were used to calculate both correlations and reference ranges for normal values of each fitted parameter as a function of age. This technique provides an age-dependent mean value and a ‘normal range' for a given parameter, accounting for the fact that both the mean and the standard deviation of the raw data may vary with age. Data were log transformed where necessary to ensure normality of the residuals, and a backwards stepwise regression method was used, starting with a second-order polynomial, then reducing the degree to a first-order polynomial (i.e., linear fit), provided a significant worsening of the fit was not observed (details in ref. 24). All reference ranges were calculated with 95% confidence intervals.

### Statistical Analysis

All comparisons between correlations were performed using an analysis of covariance (ANCOVA) test, followed by a Tukey-Kramer *post hoc* analysis (significance was defined as *P*<0.05). As part of the *post hoc* analysis, population marginal means (PMMs) were compared, which represent the predicted value of the dependent variable for the mean value of the independent variable (age in our case). Differences in PMMs are listed in the Results section in cases where significance was reached (*P*<0.05).

## Results

In all ROIs, none of the fitted parameters showed a significant difference in their correlation with age as a function hemisphere (*i.e.*, left- and right-hand sides were equivalent). Therefore, all regional values represent the mean for the left-/right-hand side of the brain. A significant difference between males and females was only seen in one parameter (*M*_0_ in the PCA territory, see *T*_1_/*M*_0_ Values section). In all other cases, a single correlation/reference range is provided, for male and female subjects combined.

### *T*_1_/*M*_0_ Values

**Figure 2:**
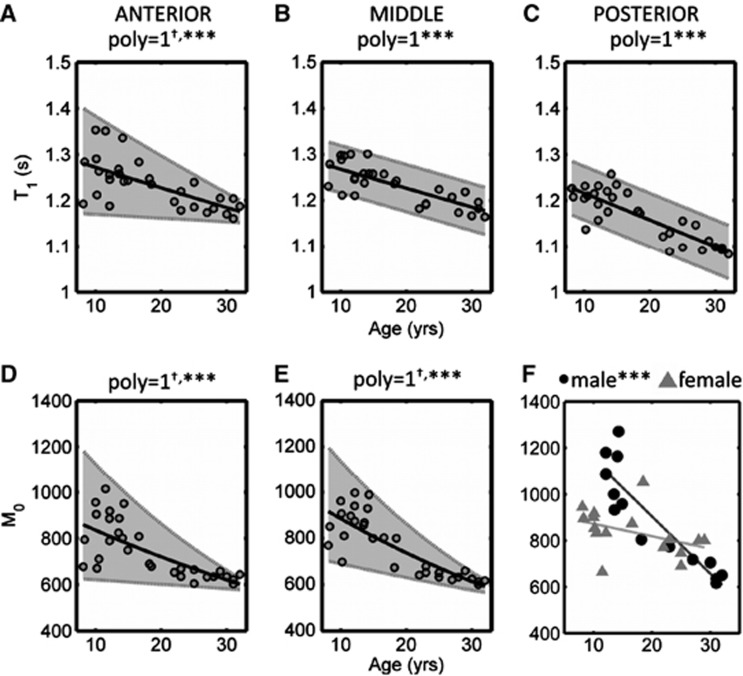
Variation in fitted values of *T*_1_ and *M*_0_ as a function of age. Data are shown for the cortical vascular territories of the anterior (left column), middle (center column), and posterior (right column) cerebral arteries. Data points represent mean values for left- and right-hand sides of the brain. Data are shown for males and females combined, except in panel **F**, in which a significant difference in the correlation between *M*_0_ vs. age was observed in males compared with females; here, individual correlations are shown (black circles=male, gray triangles=female). *T*_1_ values are shown in the top row (**A**–**C**) and *M*_0_ values are shown in the bottom row (**D**–**F**). Reference ranges are indicated in gray (for a 95% confidence level), with the fitted curve illustrated in black. The order of the polynomial fit is indicated by ‘poly' (i.e., poly=1 is linear and poly=2 is quadratic), and † indicates that the data were log-transformed before fitting. The significance of the fit is indicated by: ***P<0.001, **P<0.01, *P<0.05.

The variation in *T*_1_ and *M*_0_ as a function of age is shown in Figure 2. *T*_1_ showed a significant negative correlation with age (*P*<0.001) in all regions. The PMM was significantly lower for *T*_1_ values in the PCA territory (1.17 seconds), compared with both the MCA and ACA territories (1.23 seconds and 1.24 seconds, respectively). *M*_0_ also showed a negative correlation with age in the ACA and MCA vascular territories. The vascular territory of the PCA was one case in which a significant difference between males and females was observed. Here, males showed a highly significant negative correlation between *M*_0_ and age (*P*<0.001); however, no significant correlation was observed in females (*P*=0.11). In this case, data are shown separately for male and female subjects (Figure 2F).

### d*M*_max_/TI_max_ Values

**Figure 3:**
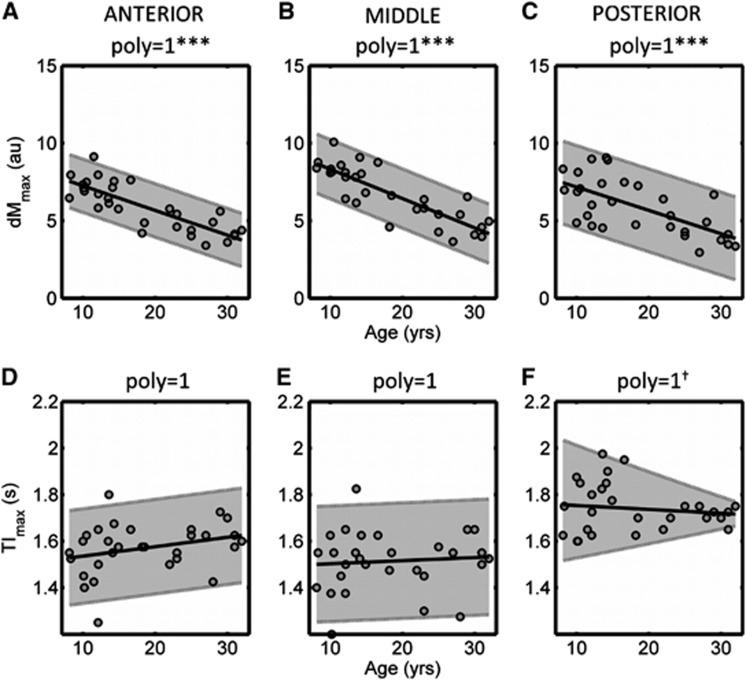
Variation in the magnitude of the peak arterial spin labeling (ASL) signal (d*M*_max_; **A**–**C**), and the inflow time (TI) at which the peak signal occurs (TI_max_, **D**–**F**), as a function of age. All symbols are identical to those used in Figure 2.

The variation in d*M*_max_ and TI_max_ as a function of age is shown in Figure 3. The magnitude of the peak ASL signal (d*M*_max_) showed a strong significant negative correlation with age in all vascular territories (*P*<0.001). In these regions, the mean d*M*_max_ value at 8 years of age was over double the mean value at the age of 30. There were no significant differences between the PMM d*M*_max_ values in all regions (*P*=0.11). The TI at which the peak occurred (TI_max_) showed no correlation with age in all vascular territories. The PMM was significantly higher on the PCA territory (1.74 seconds) than that found in both the ACA (1.57 seconds) and MCA (1.51 seconds) territories.

### Bolus Arrival Time, CBF, and *T* Values

**Figure 4:**
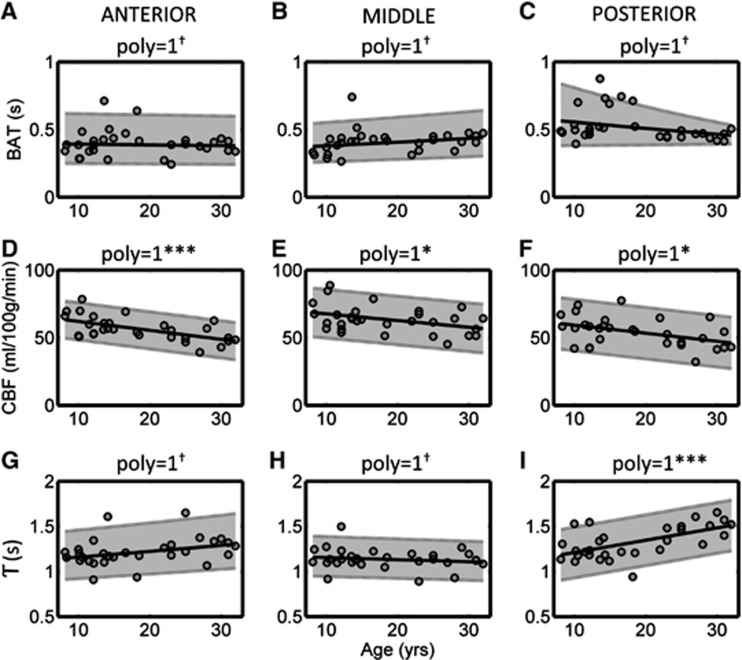
Variation in the fitted values of bolus arrival time (BAT, **A**–**C**), cerebral blood flow (CBF; **D**–**F**), and bolus duration (*T*; **G**–**I**) as a function of age. All symbols are identical to those used in Figure 2.

The variation in BAT, CBF, and T as a function of age is shown in Figure 4. Fitted values of BAT showed no correlation with age in all vascular territories, and the PMM value in the PCA (0.53 seconds) was significantly higher than that found in the ACA (0.40 seconds) and MCA (0.41 seconds) territories. Cerebral blood flow showed a negative correlation with age in all regions, which was particularly strong in the ACA territory (*P*=0.0005) compared with the MCA (*P*=0.03) and PCA (*P*=0.02) territories. The PMM value was higher in the MCA territory (63.4 mL/100 g per minute), compared with both the ACA (56.4 mL/100 g per minute) and PCA (54.2 mL/100 g per minute) territories. A significant positive correlation between age and *T* was observed in the PCA (*P*=0.0005) territory; this fell just short of significance in the ACA (*P*=0.07) territory, and no correlation was observed in the MCA territory (*P*=0.4). The PMM value of *T* was significantly higher in the PCA territory (1.32 seconds), compared with both the ACA (1.22 seconds) and MCA (1.14 seconds) territories.

The raw values for the mean curves, and upper and lower reference range limits, for all fitted parameters shown above, are given in the Supplementary Material.

**Figure 5:**
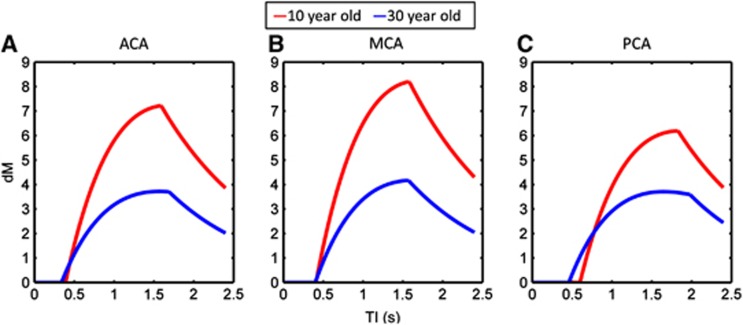
Examples of the typical dynamic arterial spin labeling (ASL) signal (d*M*) in representative healthy 10- and 30-year olds (red and blue curves, respectively). The d*M* curves were calculated using the mean, age-matched values of bolus arrival time (BAT), cerebral blood flow (CBF), *T*, *T*_1_, and *M*_0_ shown in Figures 2, 3, 4. Data are shown for the vascular territories of the anterior cerebral artery (ACA; **A**), middle cerebral artery (MCA; **B**), and posterior cerebral artery (PCA, **C**).

Examples of the dynamic ASL signal, calculated using equation (1) and incorporating the mean, age-matched values of BAT, CBF, *T*, *T*_1_, and *M*_0_, for representative 10- and 30-year olds, are shown in Figure 5. Between 10 and 30 years of age, the MCA territory showed the largest decrease in the magnitude in the ASL signal, however, the overall shape of the d*M* curves was approximately similar for the two ages. Conversely, the PCA territory showed the smallest difference in the magnitude of the ASL signal, but the d*M* curve at 30 years was much ‘wider' and ‘flatter' compared with the curve at 10 years (this was also evident, to a lesser extent, in the ACA territory). This results in a greater fitted value of *T*.

### Clinical Example in a Sturge–Weber Syndrome Patient

**Figure 6:**
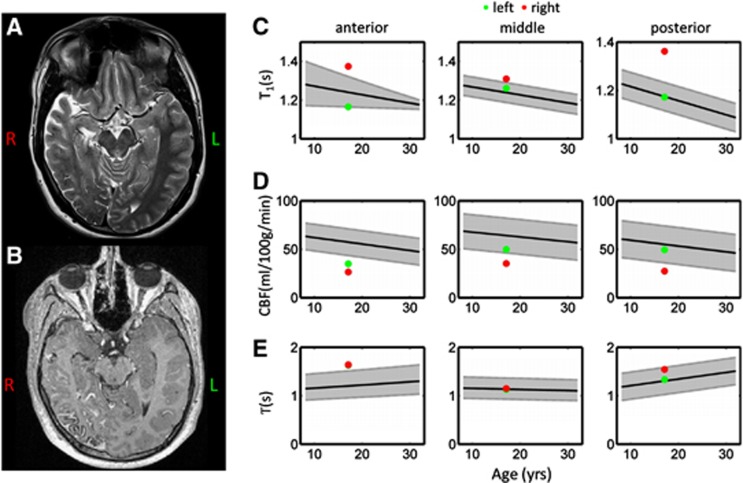
Example standard clinical images and arterial spin labeling (ASL) perfusion parameters acquired in a 17-year-old Sturge–Weber patient. Axial *T*_2_-weighted and postgadolinium *T*_1_-weighted images are shown in panels **A** and **B**, respectively. The ASL and *T*_1_-mapping parameters that fell outside the reference ranges in this patient are shown in panels **C**–**E**. The reference range acquired in healthy subjects is shown in gray in all plots, for vascular territories of the anterior- (left-hand column), middle- (center column), and posterior- (right-hand column) cerebral arteries. *T*_1_ values are shown on the top row, cerebral blood flow (CBF) on the center row, and *T* on the bottom row. Measured values in this patient are overlaid, and are shown for each hemisphere (left=green data points and right=red data points).

Figure 6 shows example *T*_2_-weighted and postgadolinium *T*_1_-weighted images for a patient with Sturge–Weber syndrome, along with the fitted ASL and T_1_-mapping parameters which fell outside the reference range regions in this patient. The *T*_2_- and *T*_1_-weighted images revealed cerebral atrophy and the corresponding leptomeningeal enhancement on the patient's right-hand side. Elevated cortical *T*_1_ values (above the reference range) were found throughout the right cerebral hemisphere (RCH), particularly in the vascular territories of the PCA and the ACA. Reduced CBF was observed throughout the brain in the RCH, and was also below the reference range in the ACA territory in the left cerebral hemisphere. Elevated values of *T* were observed bilaterally in the ACA territory.

### Clinical Example in a Neurologically Asymptomatic Sickle Cell Anemia Patient

**Figure 7:**
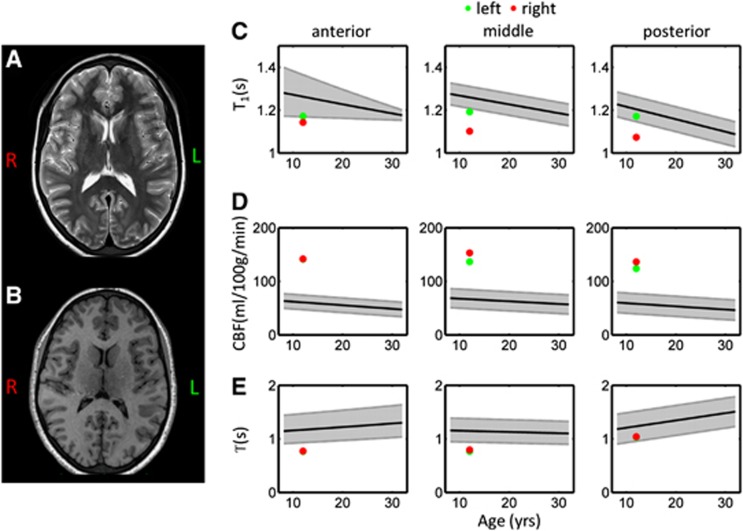
Example standard clinical images and arterial spin labeling (ASL) perfusion parameters acquired in a neurologically asymptomatic 12-year-old sickle cell anemia patient. Axial *T*_2_-weighted and *T*_1_-weighted images are shown in panels **A** and **B**, respectively. The ASL and *T*_1_-mapping parameters that fell outside the reference ranges in this patient are shown in panels **C**–**E**. All symbols are identical to those used in Figure 6.

Figure 7 shows example *T*_2_- and *T*_1_-weighted images for the SCA patient included in this study, along with the fitted ASL and T_1_-mapping parameters that fell outside the reference range regions in this patient. Two independent neuroradiologists concurred this patient has no evidence of cerebral infarction on *T*_2_-weighted MRI. Reduced cortical *T*_1_ values were observed throughout the right hemisphere, as well as on the left-hand side in the MCA territory. Very high values of CBF were observed bilaterally in all regions (142, 145, and 130 mL/100 g per minute in the vascular territories of ACA, MCA, and PCA, respectively, mean of left- and right-hand sides). These values were approximately double the average value in healthy age-matched subjects. Reduced values of *T* were observed bilaterally, in vascular territories of ACA and MCA.

## Discussion

### Healthy Subjects

Our CBF measurements in normal GM agree well with a number of previous ASL studies. For instance, in a cohort with a mean age of 11±3 years (mean±s.d.), Jain *et al*^13^ reported a mean CBF value of 65±10 mL/100 g per minute, calculated using measured, subject-specific blood T1 values, which agrees very well with our mean value at 11 years of 62±4 mL/100 g per minute (mean of all GM regions ±s.d.). At the older end of our cohort, our mean value of 50±6 mL/100 g per minute at 32 years agrees well with the study by Wang *et al*,^18^ who measured a mean value of 58±12 mL/100 g per minute in an adult group with mean age of 32 years. It should be noted however that the range of CBF values in normal GM reported in the literature varies considerably, as measured values will be influenced by factors such as the chosen TI in single-TI studies, and often fixed, adult values of blood T1 are used for data processing, which will lead to an overestimation of CBF in younger subjects (particularly girls, due to differences in hematocrit levels during development). Alternatively, positron emission tomography is considered by many to be the current standard in human CBF measurements. Considering a subset of the data presented in Leenders *et al*^25^ (including only their subjects between 22 and 32 years), using ^15^O positron emission tomography, GM CBF was measured as 59±17 mL/100 g per minute at 27±4 years, which compares well with our value of 53±5 mL/100 g per minute at the same age. Finally, Chiron *et al*,^26^ using ^133^Xe SPECT, measured mean GM CBF values of 62±10 mL/100 g per minute in the 6 to 19 years age range (no mean age is given), and 51±8 mL/100 g per minute in the 18 to 29 years range, which also agrees very well with our values.

The strong negative correlation between d*M*_max_ and age shows how certain aspects of pediatric physiology provide a natural solution to overcome the limitation of low SNR inherent in the ASL technique. The increased ASL signal at a younger age is due in part to increased blood flow in children; our CBF measurements show that between the ages of 8 to 32 years CBF showed a significant negative correlation with age in all regions. This contradicts a previous computed tomography study by Wintermark *et al*,^27^ in which regional CBF levels were shown to peak between 2 and 4 years of age, and then stabilize at adult levels at ∼7 years of age. Alternatively, using ^133^Xe single-photon emission computed tomography, Chiron *et al*^26^ measured increasing levels of CBF from birth until 5 or 6 years of age, followed by a steady decrease, reaching typical adult levels (defined as those found in 19- to 29-year olds) between 15 and 20 years (as data were averaged for subjects over 19 years in this study, later age-related changes in CBF may have been lost). Both Wintermark's and Chiron's studies involved radiation and so included children with an indication for assessment but in whom parenchymal imaging was normal, whereas ASL may be used in completely normal children. A previous ASL-based study by Taki *et al*^19^ showed a range in GM CBF trajectories between 5 and 18 years of age, varying between negative linear to cubic relationships, depending on the location. The age at which CBF peak values occurred also varied by region (from <5 years in the occipital lobe to ∼11 years in the frontal lobe). Finally, our results show the best agreement with a previous ASL study by Biagi *et al*,^2^ who showed elevated, constant CBF levels between 4 and 10 years, which then decreased rapidly during adolescence, until a plateau was reached around 25 to 30 years of age.

Our data confirm that *T*_1_ relaxation times in GM tissue show a strong negative correlation with age between 8 and 32 years. This further improves the SNR of the ASL signal in younger subjects, as following exchange at the capillaries, the longitudinal magnetization of the labeled blood water will decay according to *T*_1_ of the surrounding tissue. As this is longer in younger subjects, the effective half-life of the tracer is prolonged. The increased *T*_1B_ values at younger ages^23^ contribute further to this increase in SNR. A similar decrease in *M*_0_ with age was seen in most regions. The age-related reductions in both *T*_1_ and *M*_0_ are likely to be a result of both increased water content of the brain in children compared with adults^28^ and the onset of cortical thinning that occurs during adolescence.^29^ It is interesting that the evolution of *M*_0_ in the PCA territory is the only region in which a significant difference between males and females was seen, with males showing a significant decline in *M*_0_ with increasing age, whereas females showed no significant correlation. The data presented in ref. 29 showed that cortical thinning occurs at different rates in males and females during adolescence, depending on the location. This could be the underlying cause for the different rates of change in these parameters between males and females in the PCA territory; however, this remains speculative, as high-resolution anatomic imaging was not performed as part of this study, so GM cortical thickness could not be measured.

Previous transcranial Doppler ultrasound studies have shown a significant decline in time-averaged flow velocities (TAV) in ACA, MCA, and PCA, between children aged 10 to 17.5 years, and adults aged 20 to 63 years.^30^ Between children aged 1.5 to 9.9 years and children aged 10 to 17.5 years, no change in TAV was observed in ACA and PCA, but a decline was seen in the MCA. In general, TAV was highest in the MCA, followed by the ACA, and lowest in the PCA. However, in this study we observed no change in BAT as a function of age in all vascular territories. As a higher TAV should lead to shorter values of BAT, this would suggest that TAV is constant in all vascular territories between 8 and 32 years of age. However, these results should be interpreted with caution, as the temporal resolution of the ASL measurements made in this study is likely to be too coarse to identify small-scale changes in the underlying BAT. Furthermore, although some voxels within each ROI will include signal from large arteries branching from ACA, MCA and PCA, the majority of voxels will be distal to these feeding arteries, and hence the ASL parameters predominantly reflect blood flow in arterioles and capillaries, which will be less sensitive to changes in the TAV upstream in the feeding artery. However, the fact that that the PMM value of BAT was significantly higher in the PCA vascular territory, compared with both the MCA and ACA territories, does support previous findings that TAV is lowest in the PCA. Furthermore, our finding that CBF was significantly higher in the MCA territory compared with both the ACA and PCA territories agrees with previous evidence that TAV is highest in the MCA.

Around the age of 8 years, all three regions (ACA, MCA, and PCA territories) showed similar values of *T* (1.1 seconds in our case, although absolute values of *T* will vary depending on the acquisition protocol and the use of QUIPSS-II pulses). However, the relative change in *T* as a function of age progressed differently in each region, remaining constant in the MCA territory, while showing a highly significant increase with age in the PCA territory, and a trend (*P*=0.07) for increasing values with age in the ACA territory. As shown in Figure 5, the increased value of *T* was due to a ‘wider/flatter' d*M* curve in these regions in older subjects, particularly in the PCA territory. The increase in *T* suggests an increasing level of bolus dispersion in these territories with increasing age (note, an increase in the overall volume of tagged blood with age, due to slower blood velocities in the carotid arteries, could also be a cause of longer values of *T*, as QUIPSS-II pulses were not used in this study. However, if this was the case, then a change in *T* would have been observed in all regions). The general kinetic model used in this study makes the common assumption that the labeled bolus can be modeled using ‘plug-flow' in the vasculature, in which there is no dispersion. However, it has been shown that this is not representative of fluid dynamics in an ASL acquisition, and the labeled bolus is both pulsatile and has a time varying, nonuniform cross-sectional flow profile.^31, 32^ A number of models have been suggested to correct for this, for instance by introducing a Gaussian distribution of tag arrival times at the imaging voxel, and assuming parabolic flow in major arteries.^33^ As the temporal width of the d*M* curve seems to increase with age, particularly in the PCA territory, it would appear that the labeled blood bolus is becoming more dispersed with increasing age, and these models would therefore become particularly relevant in this vascular territory. Furthermore, this natural increase in bolus dispersion with increasing age should be accounted for when assessing changes in *T* in the presence of pathologies in future clinical studies.

### Clinical Examples

The aim of this study was to provide reference range values to describe the hemodynamic properties of the ASL signal in healthy children and young adults, which can be used as a tool in future studies to investigate pathologies that lead to abnormal cerebral perfusion. Sturge–Weber syndrome is a good example of one such pathology, characterized by intracranial venous dysplasia, a cutaneous capillary angioma, and ocular abnormalities due to anomalous embryonic development.^34^ An abnormal, tortuous pial vasculature is present, associated with venous stasis, leading to perfusion defects, which have been observed both focally in regions of leptomeningeal disease and in more distant GM in ipsilateral and contralateral regions.^35^ Clinically, there is commonly epilepsy and contralateral hemiparesis in addition to high intraocular pressure (glaucoma). The Sturge–Weber syndrome patient included in this study had a right pial angioma, with evidence of leptomeningial enhancement in the right PCA territory (identified on *T*_1_-weighted images, postcontrast), and atrophy in the RCH. *T*_1_ values were consistently higher in the RCH in all vascular territories, which may be due to the effects of edema. Cerebral blood flow was below the reference range in all regions of the RCH, and also in the left ACA territory. This illustrates how hypoperfusion can be observed both locally and remote to regions of leptomeningial disease, and also the importance of accounting for age when considering CBF values. If this patient's CBF values had been compared with healthy 30-year-old adults, then the ACA and PCA values would appear within the normal range, however, the values are low for a 17-year-old patient. The decreased values of CBF are likely to be due to impaired venous clearance in these regions. The elevated values of *T* bilaterally in the ACA territory may be representative of regions of increased tortuosity in the vasculature. It is interesting that this occurs in regions contralateral to leptomeningial enhancement; previous studies have shown perfusion abnormalities before the onset of ‘structural' abnormalities indicated by conventional imaging,^36^ which may be the case in this patient. Follow-up imaging would be required to confirm this.

Sickle cell anemia is another pathology in which the reference range tool we have developed would be particularly useful. It is a genetic condition that results in the production of abnormal hemoglobin and subsequent chronic hemolytic anemia. As a compensatory mechanism for low hematocrit, both high cerebral blood velocity and flow have been described.^37, 38, 39^ Children with SCA are at high risk of both clinical and silent cerebral infarction, narrowing of large vessels, and insufficient oxygen/glucose delivery, which may result in deficits in brain function even in patients without any abnormality seen on *T*_2_-weighted MRI. The SCA patient included in this study was one such case, in which hemoglobin and hematocrit levels were below the normal range, but no evidence of infarction was visible on the *T*_2_-weighted images. However, CBF was well above the age-matched reference range in all vascular territories in this patient, being approximately double the expected value in a typical 12-year-old patient. This is indicative of a compensatory increase in blood flow due to the low hematocrit in this patient (it should be noted that quoted values of CBF in this patient may be slightly inaccurate, as the literature value of *T*_1B_ used does not account for the low hematocrit in this patient). Similarly, the short values of *T* observed in the ACA and MCA territories are likely to be due to increased blood flow velocities in the feeding arteries, resulting in a reduction in the level of bolus dispersion. Reduced values of *T*_1_ were observed in all vascular territories on the RHS, and bilaterally in the MCA territory. This agrees with a previous study, which showed reduced values of *T*_1_ in the GM of SCA patients, even in the scans of patients appearing normal on conventional MRI.^40^ It has been suggested that this is indicative of brain damage due to moderate hypoxia, and is particularly present in tissues with a higher metabolic rate,^16^ which could explain the reduction in GM *T*_1_ values seen in this patient.

## Conclusion

Overall, our results show that during the transition from childhood into adulthood, significant changes occur in both *T*_1_ and *M*_0_ of cortical GM and the hemodynamic properties of CBF. Due to the strong age dependence of *T*_1_ and *M*_0_, if assumed rather than measured values are used when calculating CBF from raw ASL data (as is often the case in clinical studies, when scan time is limited), age-corrected values should be used, as adopting adult values of *M*_0_ and *T*_1_ in equation (1) will lead to a significant overestimation of CBF in children. Similarly, once CBF values have been calculated, the data should be compared with age-matched reference range values, due to the normal variation with age in healthy subjects in this age range. In multi-TI ASL studies, normal values of *T* will also vary as a function of age (depending on the vascular region), which should be accounted for when designing a protocol to investigate pathologies in this region.

## References

[bib1] Gogtay N Giedd JN Lusk L Hayashi KM Greenstein D Vaituzis AC Dynamic mapping of human cortical development during childhood through early adulthood Proc Natl Acad Sci USA 2004 101 8174 8179 15148381 10.1073/pnas.0402680101 PMC419576

[bib2] Biagi L Abbruzzese A Bianchi MC Alsop DC Del Guerra A Tosetti M Age dependence of cerebral perfusion assessed by magnetic resonance continuous arterial spin labeling J Magn Reson Imaging 2007 25 696 702 17279531 10.1002/jmri.20839

[bib3] Sokoloff L Reivich M Kennedy C Rosiers MHD Patlak CS Pettigrew KD The [14c]deoxyglucose method for the measurement of local cerebral glucose utilization: theory, procedure, and normal values in the conscious and anesthetized Albino rat J Neurochem 1977 28 897 916 864466 10.1111/j.1471-4159.1977.tb10649.x

[bib4] Broome DR Nephrogenic systemic fibrosis associated with gadolinium based contrast agents: a summary of the medical literature reporting Eur J Radiol 2008 66 230 234 18372138 10.1016/j.ejrad.2008.02.011

[bib5] Detre JA Leigh JS Williams DS Koretsky AP Perfusion imaging Magn Reson Med 1992 23 37 45 1734182 10.1002/mrm.1910230106

[bib6] Williams DS Detre JA Leigh JS Koretsky AP Magnetic resonance imaging of perfusion using spin inversion of arterial water Proc Natl Acad Sci USA 1992 89 212 216 1729691 10.1073/pnas.89.1.212 PMC48206

[bib7] Chen T-Y Chiu L Wu T-C Wu T-C Lin C-J Wu S-C Arterial spin-labeling in routine clinical practice: a preliminary experience of 200 cases and correlation with MRI and clinical findings Clin Imaging 2012 36 345 352 22726973 10.1016/j.clinimag.2011.11.003

[bib8] Golay X Guenther M Arterial spin labelling: final steps to make it a clinical reality MAGMA 2012 25 79 82 22382350 10.1007/s10334-012-0308-9

[bib9] Wong EC Buxton RB Frank LR Implementation of quantitative perfusion imaging techniques for functional brain mapping using pulsed arterial spin labeling NMR Biomed 1997 10 237 249 9430354 10.1002/(sici)1099-1492(199706/08)10:4/5<237::aid-nbm475>3.0.co;2-x

[bib10] Buxton RB Frank LR Wong EC Siewert B Warach S Edelman RR A general kinetic model for quantitative perfusion imaging with arterial spin labeling Magn Reson Med 1998 40 383 396 9727941 10.1002/mrm.1910400308

[bib11] Luh W-M Wong EC Bandettini PA Hyde JS QUIPSS II with thin-slice TI periodic saturation: A method for improving accuracy of quantitative perfusion imaging using pulsed arterial spin labeling Magn Reson Med 1999 41 1246 1254 10371458 10.1002/(sici)1522-2594(199906)41:6<1246::aid-mrm22>3.0.co;2-n

[bib12] Herscovitch P Raichle ME What is the correct value for the brain-blood partition coefficient for water J Cereb Blood Flow Metab 1985 5 65 69 3871783 10.1038/jcbfm.1985.9

[bib13] Jain V Duda J Avants B Giannetta M Xie SX Roberts T Longitudinal reproducibility and accuracy of pseudo-continuous arterial spin–labeled perfusion MR imaging in typically developing children Radiology 2012 263 527 536 22517961 10.1148/radiol.12111509 PMC3329270

[bib14] Wu W-C Jain V Li C Giannetta M Hurt H Wehrli FW *In vivo* venous blood T1 measurement using inversion recovery true-FISP in children and adults Magn Reson Med 2010 64 1140 1147 20564586 10.1002/mrm.22484 PMC2946493

[bib15] Ye FQ Pekar JJ Jezzard P Duyn J Frank JA McLaughlin AC Perfusion imaging of the human brain at 1.5T using a single-shot EPI spin tagging approach Magn Reson Med 1996 36 217 224 8843375 10.1002/mrm.1910360208

[bib16] Steen RG Langston JW Ogg RJ Xiong X Ye Z Wang WC Diffuse T1 reduction in gray matter of sickle cell disease patients: evidence of selective vulnerability to damage Magn Reson Imaging 1999 17 503 515 10231177 10.1016/s0730-725x(98)00204-5

[bib17] Parkes LM Rashid W Chard DT Tofts PS Normal cerebral perfusion measurements using arterial spin labeling: reproducibility, stability, and age and gender effects Magn Reson Med 2004 51 736 743 15065246 10.1002/mrm.20023

[bib18] Wang J Licht DJ Jahng G-H Liu C-S Rubin JT Haselgrove J Pediatric perfusion imaging using pulsed arterial spin labeling J Magn Reson Imaging 2003 18 404 413 14508776 10.1002/jmri.10372

[bib19] Taki Y Hashizume H Sassa Y Takeuchi H Wu K Asano M Correlation between gray matter density-adjusted brain perfusion and age using brain MR images of 202 healthy children Hum Brain Mapp 2011 32 1973 1985 21259384 10.1002/hbm.21163 PMC6870039

[bib20] Zoccoli G Lucchi ML Andreoli E Bach V Cianci T Lenzi P Brain capillary perfusion during sleep J Cereb Blood Flow Metab 1996 16 1312 1318 8898706 10.1097/00004647-199611000-00028

[bib21] Günther M Oshio K Feinberg DA Single-shot 3D imaging techniques improve arterial spin labeling perfusion measurements Magn Reson Med 2005 54 491 498 16032686 10.1002/mrm.20580

[bib22] Tatu L Moulin T Bogousslavsky J Duvernoy H Arterial territories of the human brain cerebral hemispheres Neurology 1998 50 1699 1708 9633714 10.1212/wnl.50.6.1699

[bib23] Piechnik SK Ferreira VM Lewandowski AJ Ntusi NA Banerjee R Holloway C Normal variation of magnetic resonance T1 relaxation times in the human population at 1.5T using ShMOLLI J Cardiovasc Magn Reson 2013 15 13 23331520 10.1186/1532-429X-15-13 PMC3610210

[bib24] Royston P Constructing time-specific reference ranges Stat Med 1991 10 675 690 2068420 10.1002/sim.4780100502

[bib25] Leenders KL Perani D Lammertsma AA Heather JD Buckingham P Jones T Cerebral blood flow, blood volume and oxygen utilization Brain 1990 113 27 47 2302536 10.1093/brain/113.1.27

[bib26] Chiron C Raynaud C Maziere B Zilbovicius M Laflamme L Masure M-C Changes in regional cerebral blood flow during brain maturation in children and adolescents J Nucl Med 1992 33 696 703 1569478

[bib27] Wintermark M Lepori D Cotting J Roulet E van Melle G Meuli R Brain perfusion in children: evolution with age assessed by quantitative perfusion computed tomography Pediatrics 2004 113 1642 1652 15173485 10.1542/peds.113.6.1642

[bib28] Dobbing J Sands J Quantitative growth and development of human brain Arch Dis Child 1973 48 757 767 4796010 10.1136/adc.48.10.757 PMC1648530

[bib29] Raznahan A Lee Y Stidd R Long R Greenstein D Clasen L Longitudinally mapping the influence of sex and androgen signaling on the dynamics of human cortical maturation in adolescence Proc Natl Acad Sci USA 2010 107 16988 16993 20841422 10.1073/pnas.1006025107 PMC2947865

[bib30] Schöning M Staab M Walter J Niemann G Transcranial color duplex sonography in childhood and adolescence. Age dependence of flow velocities and waveform parameters Stroke 1993 24 1305 1309 8362422 10.1161/01.str.24.9.1305

[bib31] Tortoli P Michelassi V Bambi G Guidi F Righi D Interaction between secondary velocities, flow pulsation and vessel morphology in the common carotid artery Ultrasound Med Biol 2003 29 407 415 12706192 10.1016/s0301-5629(02)00705-6

[bib32] Wu W-C Mazaheri Y Wong EC The effects of flow dispersion and cardiac pulsation in arterial spin labeling IEEE Trans Med Imaging 2007 26 84 92 17243587 10.1109/TMI.2006.886807

[bib33] Gallichan D Jezzard P Modeling the effects of dispersion and pulsatility of blood flow in pulsed arterial spin labeling Magn Reson Med 2008 60 53 63 18581416 10.1002/mrm.21654

[bib34] Comi AM Pathophysiology of Sturge-Weber syndrome J Child Neurol 2003 18 509 516 13677575 10.1177/08830738030180080701

[bib35] Evans AL Widjaja E Connolly DJA Griffiths PD Cerebral perfusion abnormalities in children with Sturge-Weber syndrome shown by dynamic contrast bolus magnetic resonance perfusion imaging Pediatrics 2006 117 2119 2125 16740855 10.1542/peds.2005-1815

[bib36] Reid DE Maria BL Drane WE Quisling RG Hoang KB Central nervous system perfusion and metabolism abnormalities in Sturge-Weber syndrome J Child Neurol 1997 12 218 222 9130099 10.1177/088307389701200313

[bib37] Kirkham FJ Calamante F Bynevelt M Gadian DG Evans JP Cox TC Perfusion magnetic resonance abnormalities in patients with sickle cell disease Ann Neurol 2001 49 477 485 11310625

[bib38] Oguz KK Golay X Pizzini FB Freer CA Winrow N Ichord R Sickle cell disease: continuous arterial spin-labeling perfusion MR imaging in children Radiology 2003 227 567 574 12663827 10.1148/radiol.2272020903

[bib39] Prohovnik I Hurlet-Jensen A Adams R De Vivo D Pavlakis SG Hemodynamic etiology of elevated flow velocity and stroke in sickle-cell disease J Cereb Blood Flow Metab 2009 29 803 810 19209182 10.1038/jcbfm.2009.6

[bib40] Steen RG Schroeder J Age-related changes in the pediatric brain: proton T1 in healthy children and in children with sickle cell disease Magn Reson Imaging 2003 21 9 15 12620541 10.1016/s0730-725x(02)00635-5

