# Supplementary material for: Arterial spin labeling characterization of cerebral perfusion during normal maturation from late childhood into adulthood: normal ‘reference range' values and their use in clinical studies
Source: J Cereb Blood Flow Metab. 2014 Feb 5;34(5):776–84. doi: 10.1038/jcbfm.2014.17 (PMC4013758; doi:10.1038/jcbfm.2014.17)
Supplement: Supplementary Materials [file jcbfm201417x1.doc]

**Supplementary Material**

Tables S1,S2, and S3 below give the raw values for the mean curves, and upper and lower reference range limits, for the fitted parameters shown in Figures 2-4.

**Table S1 ACA territory**

| Age | T1 low | T1 | T1 upp | M0 low | M0 | M0 upp | BAT low | BAT | BAT upp | CBF low | CBF | CBF upp | Tau low | Tau | Tau upp |
| --- | --- | --- | --- | --- | --- | --- | --- | --- | --- | --- | --- | --- | --- | --- | --- |
| 8 | 1.17 | 1.28 | 1.40 | 624.29 | 860.35 | 1185.67 | 0.17 | 0.40 | 0.63 | 52.26 | 69.98 | 87.70 | 0.86 | 1.17 | 1.59 |
| 10 | 1.17 | 1.27 | 1.38 | 620.31 | 835.18 | 1124.48 | 0.17 | 0.40 | 0.63 | 50.48 | 68.20 | 85.92 | 0.89 | 1.18 | 1.58 |
| 12 | 1.17 | 1.26 | 1.37 | 616.36 | 810.75 | 1066.44 | 0.16 | 0.39 | 0.62 | 48.71 | 66.42 | 84.14 | 0.92 | 1.20 | 1.57 |
| 14 | 1.17 | 1.25 | 1.35 | 612.43 | 787.03 | 1011.40 | 0.16 | 0.39 | 0.62 | 46.93 | 64.65 | 82.36 | 0.95 | 1.21 | 1.56 |
| 16 | 1.16 | 1.25 | 1.33 | 608.53 | 764.00 | 959.20 | 0.15 | 0.38 | 0.61 | 45.15 | 62.87 | 80.59 | 0.98 | 1.23 | 1.55 |
| 18 | 1.16 | 1.24 | 1.31 | 604.65 | 741.65 | 909.70 | 0.15 | 0.38 | 0.61 | 43.37 | 61.09 | 78.81 | 1.01 | 1.25 | 1.54 |
| 20 | 1.16 | 1.23 | 1.30 | 600.80 | 719.96 | 862.75 | 0.15 | 0.37 | 0.60 | 41.59 | 59.31 | 77.03 | 1.04 | 1.26 | 1.53 |
| 22 | 1.16 | 1.22 | 1.28 | 596.97 | 698.89 | 818.22 | 0.14 | 0.37 | 0.60 | 39.81 | 57.53 | 75.25 | 1.07 | 1.28 | 1.52 |
| 24 | 1.16 | 1.21 | 1.26 | 593.16 | 678.45 | 775.99 | 0.14 | 0.36 | 0.59 | 38.04 | 55.75 | 73.47 | 1.11 | 1.29 | 1.51 |
| 26 | 1.16 | 1.20 | 1.25 | 589.38 | 658.60 | 735.94 | 0.13 | 0.36 | 0.59 | 36.26 | 53.98 | 71.69 | 1.14 | 1.31 | 1.50 |
| 28 | 1.16 | 1.19 | 1.23 | 585.63 | 639.33 | 697.96 | 0.13 | 0.36 | 0.58 | 34.48 | 52.20 | 69.92 | 1.18 | 1.33 | 1.50 |
| 30 | 1.15 | 1.18 | 1.22 | 581.90 | 620.63 | 661.94 | 0.12 | 0.35 | 0.58 | 32.70 | 50.42 | 68.14 | 1.22 | 1.34 | 1.49 |
| 32 | 1.15 | 1.18 | 1.20 | 578.19 | 602.47 | 627.77 | 0.12 | 0.35 | 0.58 | 30.92 | 48.64 | 66.36 | 1.25 | 1.36 | 1.48 |

**Table S2 MCA territory**

| Age | T1 low | T1 | T1 upp | M0 low | M0 | M0 upp | BAT low | BAT | BAT upp | CBF low | CBF | CBF upp | Tau low | Tau | Tau upp |
| --- | --- | --- | --- | --- | --- | --- | --- | --- | --- | --- | --- | --- | --- | --- | --- |
| 8 | 1.22 | 1.28 | 1.33 | 701.85 | 918.21 | 1201.27 | 0.17 | 0.38 | 0.62 | 55.88 | 75.09 | 94.30 | 0.99 | 1.19 | 1.42 |
| 10 | 1.22 | 1.27 | 1.32 | 689.31 | 885.21 | 1136.78 | 0.18 | 0.39 | 0.62 | 54.61 | 73.82 | 93.03 | 0.99 | 1.19 | 1.42 |
| 12 | 1.21 | 1.26 | 1.31 | 676.99 | 853.39 | 1075.74 | 0.18 | 0.39 | 0.62 | 53.35 | 72.55 | 91.76 | 0.99 | 1.18 | 1.42 |
| 14 | 1.20 | 1.25 | 1.30 | 664.90 | 822.71 | 1017.99 | 0.18 | 0.39 | 0.62 | 52.08 | 71.28 | 90.49 | 0.99 | 1.18 | 1.41 |
| 16 | 1.19 | 1.24 | 1.29 | 653.02 | 793.14 | 963.33 | 0.19 | 0.40 | 0.63 | 50.81 | 70.01 | 89.22 | 0.99 | 1.18 | 1.41 |
| 18 | 1.18 | 1.23 | 1.29 | 641.35 | 764.63 | 911.61 | 0.19 | 0.40 | 0.63 | 49.54 | 68.74 | 87.95 | 0.98 | 1.18 | 1.41 |
| 20 | 1.18 | 1.23 | 1.28 | 629.89 | 737.15 | 862.67 | 0.19 | 0.40 | 0.63 | 48.27 | 67.47 | 86.68 | 0.98 | 1.18 | 1.40 |
| 22 | 1.17 | 1.22 | 1.27 | 618.64 | 710.65 | 816.35 | 0.19 | 0.41 | 0.64 | 47.00 | 66.21 | 85.41 | 0.98 | 1.17 | 1.40 |
| 24 | 1.16 | 1.21 | 1.26 | 607.58 | 685.11 | 772.52 | 0.20 | 0.41 | 0.64 | 45.73 | 64.94 | 84.14 | 0.98 | 1.17 | 1.40 |
| 26 | 1.15 | 1.20 | 1.25 | 596.73 | 660.48 | 731.05 | 0.20 | 0.41 | 0.64 | 44.46 | 63.67 | 82.87 | 0.98 | 1.17 | 1.40 |
| 28 | 1.14 | 1.19 | 1.25 | 586.07 | 636.74 | 691.80 | 0.20 | 0.41 | 0.65 | 43.19 | 62.40 | 81.60 | 0.98 | 1.17 | 1.39 |
| 30 | 1.13 | 1.19 | 1.24 | 575.60 | 613.86 | 654.66 | 0.20 | 0.42 | 0.65 | 41.92 | 61.13 | 80.34 | 0.97 | 1.16 | 1.39 |
| 32 | 1.13 | 1.18 | 1.23 | 565.31 | 591.79 | 619.51 | 0.21 | 0.42 | 0.65 | 40.65 | 59.86 | 79.07 | 0.97 | 1.16 | 1.39 |

Table S3 PCA territory

| Age | T1 low | T1 | T1 upp | M0 male | M0 female | BAT low | BAT | BAT upp | CBF low | CBF | CBF upp | Tau low | Tau | Tau upp |
| --- | --- | --- | --- | --- | --- | --- | --- | --- | --- | --- | --- | --- | --- | --- |
| 8 | 1.17 | 1.23 | 1.29 | 1201.68 | 883.59 | 0.40 | 0.56 | 0.80 | 44.96 | 67.83 | 90.70 | 1.02 | 1.23 | 1.47 |
| 10 | 1.16 | 1.22 | 1.27 | 1152.19 | 872.83 | 0.39 | 0.55 | 0.78 | 43.53 | 66.40 | 89.27 | 1.04 | 1.25 | 1.49 |
| 12 | 1.15 | 1.20 | 1.26 | 1102.71 | 862.08 | 0.39 | 0.55 | 0.77 | 42.11 | 64.98 | 87.85 | 1.06 | 1.27 | 1.52 |
| 14 | 1.13 | 1.19 | 1.25 | 1053.22 | 851.32 | 0.38 | 0.54 | 0.76 | 40.68 | 63.55 | 86.42 | 1.08 | 1.30 | 1.55 |
| 16 | 1.12 | 1.18 | 1.24 | 1003.73 | 840.56 | 0.38 | 0.53 | 0.75 | 39.26 | 62.13 | 85.00 | 1.10 | 1.32 | 1.58 |
| 18 | 1.11 | 1.17 | 1.23 | 954.25 | 829.80 | 0.37 | 0.52 | 0.74 | 37.83 | 60.70 | 83.57 | 1.12 | 1.34 | 1.61 |
| 20 | 1.10 | 1.16 | 1.22 | 904.76 | 819.04 | 0.36 | 0.51 | 0.73 | 36.41 | 59.28 | 82.15 | 1.14 | 1.37 | 1.64 |
| 22 | 1.09 | 1.15 | 1.20 | 855.28 | 808.28 | 0.36 | 0.51 | 0.72 | 34.99 | 57.86 | 80.73 | 1.17 | 1.39 | 1.67 |
| 24 | 1.08 | 1.13 | 1.19 | 805.79 | 797.52 | 0.35 | 0.50 | 0.70 | 33.56 | 56.43 | 79.30 | 1.19 | 1.42 | 1.70 |
| 26 | 1.06 | 1.12 | 1.18 | 756.31 | 786.76 | 0.35 | 0.49 | 0.69 | 32.14 | 55.01 | 77.88 | 1.21 | 1.45 | 1.73 |
| 28 | 1.05 | 1.11 | 1.17 | 706.82 | 776.00 | 0.34 | 0.48 | 0.68 | 30.71 | 53.58 | 76.45 | 1.23 | 1.47 | 1.76 |
| 30 | 1.04 | 1.10 | 1.16 | 657.33 | 765.24 | 0.34 | 0.48 | 0.67 | 29.29 | 52.16 | 75.03 | 1.26 | 1.50 | 1.80 |
| 32 | 1.03 | 1.09 | 1.15 | 607.85 | 754.48 | 0.33 | 0.47 | 0.66 | 27.86 | 50.73 | 73.60 | 1.28 | 1.53 | 1.83 |
